# Genome-Wide Association Mapping of Late Blight Tolerance Trait in Potato (*Solanum tuberosum* L.)

**DOI:** 10.3389/fgene.2021.714575

**Published:** 2021-10-01

**Authors:** Fang Wang, Meiling Zou, Long Zhao, Zhiqiang Xia, Jian Wang

**Affiliations:** ^1^Academy of Agriculture and Forestry Sciences, Qinghai University, Xining, China; ^2^National Key Laboratory of Sanjiangyuan Ecology and Plateau Agriculture and Animal Husbandry, Qinghai University, Xining, China; ^3^Key Laboratory of Qinghai-Tibet Plateau Biotechnology Ministry of Education, Xining, China; ^4^Qinghai Provincial Key Laboratory of Potato Breeding, Xining, China; ^5^College of Tropical Crops, Hainan University, Haikou, China; ^6^Institute of Tropical Biosciences and Biotechnology, Chinese Academy of Tropical Agriculture Sciences, Haikou, China

**Keywords:** *Solanum tuberosum* L., single-nucleotide polymorphism (SNP), genome-wide association analysis (GWAS), population structure, genetic diversity, late blight tolerance

## Abstract

Uncovering the genetic basis and optimizing the late blight tolerance trait in potatoes (*Solanum tuberosum* L.) are crucial for potato breeding. Late blight disease is one of the most significant diseases hindering potato production. The traits of late blight tolerance were evaluated for 284 potato cultivars to identify loci significantly associated with the late blight tolerance trait. Of all, 37 and 15 were the most tolerant to disease, and 107 and 30 were the most susceptible. A total of 22,489 high-quality single-nucleotide polymorphisms and indels were identified in 284 potato cultivars. All the potato cultivars were clustered into eight subgroups using population structure analysis and principal component analysis, which were consistent with the results of the phylogenetic tree analysis. The average genetic diversity for all 284 potato cultivars was 0.216, and the differentiation index of each subgroup was 0.025–0.149. Genome-wide linkage disequilibrium (LD) analysis demonstrated that the average LD was about 0.9 kb. A genome-wide association study using a mixed linear model identified 964 loci significantly associated with the late blight tolerance trait. Fourteen candidate genes for late blight tolerance traits were identified, including genes encoding late blight tolerance protein, chitinase 1, cytosolic nucleotide-binding site–leucine-rich repeat tolerance protein, protein kinase, ethylene-responsive transcription factor, and other potential plant tolerance-related proteins. This study provides novel insights into the genetic architecture of late blight tolerance traits and will be helpful for late blight tolerance in potato breeding.

## Introduction

Potato (*Solanum tuberosum* L.) is a major food crop produced worldwide (Paul et al., 2012). Potato productivity is largely limited by late blight disease, a typical disease that often occurs at low temperatures and high humidity, caused by *Phytophthora infestans* (Fry et al., 2002). *Phytophthora infestans* propagate asexually or sexually and invade the potato cells by hyphae, rapidly causing dead leaves and rotten tubers (McDonald and Linde, 2002; Zwankhuizen and Zadoks, 2002; Enciso-Rodriguez et al., 2008; Wu et al., 2016). In traditional agriculture, late blight is primarily controlled using protective agents or systemic fungicides. However, pesticides pollute the environment and accelerate the development of tolerance and the mutation of *Phytophthora infestans* (Maurice et al., 2009).

Risch and Merikangas (1996) and Hansen et al. (2001) first reported genome-wide association analysis (GWAS). With the development of high-throughput genotyping, particularly the genotyping sequencing (GBS) technique and amplified-fragment single-nucleotide polymorphism (SNP) and methylation (AFSM) technology, GWAS has become a powerful approach for genetic dissection of complex quantitative traits by locating quantitative trait loci affecting phenotype, using sufficient markers and linkage disequilibrium (LD) between alleles (Nordborg and Weigel, 2008; Zhu et al., 2008; Korte and Farlow, 2013; Pradhan et al., 2016; Zhang et al., 2018). Nowadays, GWAS is an efficient and reliable tool for deciphering the molecular basis of complex quantitative traits, such as pathogen tolerance in *Arabidopsis* (Aranzana et al., 2005), preharvest sprouting resistance in wheat (Zhou et al., 2017), and head smut tolerance in maize (Wang et al., 2012). In this study, we conducted GWAS for late blight tolerance traits using AFSM on 284 potato cultivars from China, Australia, Belarus, Canada, Britain, the International Potato Center (CIP), Israel, Netherlands, Russia, and the United States. Our objective was to dissect their genetic architecture, evaluate genetic diversity, identify loci, and molecular markers associated with the late blight tolerance trait, and identify candidate genes for potato-breeding improvement. Two randomly selected candidate genes were verified by qRT-PCR.

## Materials and Methods

### Sample Collection

A total of 284 potato accessions, collected from the Qinghai Plateau Potato Experimental Station, were used for GWAS to dissect the genetic basis of late blight tolerance. Among them, 97 accessions originated from China, 1 from Australia, 3 from Belarus, 5 from Canada, 5 from Britain, 138 from CIP, 2 from Israel, 1 from the Netherlands, 5 from Russia, 1 from the United States, and 26 from unknown regions (Supplementary Table 1).

All 284 potato accessions used in this study were tetraploid and were grown in the Qinghai Plateau Potato Experimental Station from the Academy of Agriculture and Forestry Sciences, Qinghai University (36°68′N, 101°26′E) in 2018 and 2019 with 10 replicates of each accession in one row.

### Phenotype Evaluation and Statistics

#### Preparation of Pathogen

The *Phytophthora infestans* used in this study were collected from different potato planting areas in Qinghai Province with physiologically dominant species, mainly 3, 4, and 10. The susceptible potatoes were cut into slices, approximately 0.5-cm thick. These slices were inoculated with the pathogen, placed in culture dishes in an 18°C incubator, incubated in the dark, and covered with moist filter papers. After 5 days, the collected sporangia on these slices were filtered using nylon and steel mesh. The concentration of *Phytophthora infestans* was adjusted to 50,000 per milliliter.

#### Indoor Inoculation and Identification

Ten leaves for each accession were picked for *in vitro* inoculation with *Phytophthora infestans* after the seedlings had 10 leaves. Each picked leaf of the 284 potato accessions was inoculated with 15 μL of *Phytophthora infestans* and placed in an 18°C humidified incubator with 8 h darkness and 16 h light every day. Then, the indoor investigation for late blight tolerance in potatoes was evaluated (Table 1) after inoculation for 5 days in 2018 and 2019. Each trait was scored on 10 leaves in each accession, and the mean of two replicates was used for subsequent statistical analysis and GWAS.

**TABLE 1:** Identification criteria for late blight tolerance of potato leaves *in vitro*.

Grade of disease	Type	Degree of morbidity
1	Most tolerance	The lesion area is less than 3% or asymptomatic
2	More tolerance	The area of the lesion was 3–10%, forming an anaphylactic necrotic spot without chlorotic halo
3	Moderately tolerance	The area of the lesion was 10–30%. The lesion was water-soaked and surrounded by chlorotic halo. White mycelium could be seen on the surface of the lesion
4	Moderately susceptible	The lesion area ranged from 30 to 60%, there was an obvious chlorotic ring and white mold layer around the lesion
5	More susceptible	The lesion area was more than 60%, and a large number of white molds appeared on the lesion surface

### DNA Preparation and Sequencing

The improved cetyltrimethylammonium bromide (CTAB) method (Murray and Thompson, 1980) was used to extract genomic DNA from potato leaves. After detecting and quantifying the concentration by 1% agarose gel electrophoresis, the working DNA solution was diluted to 100 ng/μL and stored at −20°C. The AFSM approach (Xia et al., 2014) was then used to construct EcoRI-MspI and EcoRI-HpaII libraries of 284 potato DNA samples. After the monoclonal detection met the requirements, the EcoRI-MspI and EcoRI-HpaII libraries were mixed into one library at a ratio of 1:1, and HiSeq 2500 was used to perform paired-end 150-bp sequencing on the constructed sequencing library. Out of 355 Gb of total sequencing data, 328 Gb of clean data were obtained with more than 1 Gb of data per sample.

### Single-Nucleotide Polymorphism Calling and Annotation

We used a Perl script^1^ to filter the original sequencing data, count the total number of reads obtained from sequencing, assign the reads to each individual based on the barcodes designed using the AFSM technology, and count the number of reads in each individual. Bowtie2 software (Langmead and Salzberg, 2012) aligned the optimized sequencing reads to the potato DM reference genome,^2^ and SAMtools (Li et al., 2009) and VCFtools^3^ were used to detect SNP and indel loci. Based on the potato DM reference genome v.4.03, the snpEff software (Cingolani et al., 2012) was used to identify the mutation locations (intergenic region, untranslated region/UTR, upstream gene region, or downstream gene region), mutation types (synonymous, missense, frameshift, and non-frameshift), and annotate them simultaneously.

### Analysis of Population Structure and Genetic Diversity

We first used PHYLIP^4^ to calculate the genetic distance matrix of the sample. We then used the Notepad++ software to save the genetic distance matrix file in a suitable format. A phylogenetic tree was constructed using the neighbor-joining method. After generating the tree file, iTOL^5^ was used to draw the phylogenetic tree diagram. GCTA software was used to conduct principal component analysis (PCA) of the potato population materials with the detected SNPs as inputs (Yang et al., 2011). R software was then used to calculate the vector of each principal component and draw the PCA scatter plot. Additionally, ADMIXTURE software (Alexander et al., 2009) analyzed the population structure and estimated the optimal number of population subgroups. PLINK software (Purcell et al., 2007) was used to adjust the input file format for ADMIXTURE software, and then we input the file. The subgroups’ *K*-value range was set to 1–12. The appropriate value of K for the number of subgroups was determined according to the obtained cross-validation error value. The genetic composition coefficient (Q) of each material in each subgroup was used to construct the population-genetic structure matrix. VCFtools software^6^ was used to calculate the genetic diversity (π) and population pairwise *F*-statistics (*F*_*ST*_) (Danecek et al., 2011). According to Wright, when *F*_*ST*_ is equal to zero or one, it indicates no differentiation or complete differentiation between subgroups, respectively. If 0 < *F*_*ST*_ < 0.05, 0.05 ≤ *F*_*ST*_ < 0.15, 0.15 ≤ *F*_*ST*_ < 0.25, or 0.25 ≤ *F*_*ST*_ < 1, this indicates that the subgroups have weak, medium, strong, or very strong genetic differentiation, respectively (Dunia et al., 2011). In the entire group and each subgroup (determined by the population structure), the *r*^2^-value was used to determine the genome-wide LD through pairwise comparisons between 22,489 SNP markers.

### Linkage Disequilibrium Analysis

In the entire population and each subgroup (inferred using ADMIXTURE), the value of *R*^2^ was used to evaluate the LD relationship between each pair of polymorphic sites throughout the genome, and the value of *R*^2^ was calculated using PopLDdecay software (Zhang et al., 2019) for high-quality SNPs after filtering. The genetic distance was sorted from small to large, and then the average value of LD *R*^2^ in the segment was calculated to draw a scatterplot with a smooth curve. The genetic distance interval in which the curve intersects with the straight line representing a non-collinear *R*^2^ of 99% is the LD-decay distance.

### Association Analysis

In this study, data from 22,489 high-quality SNPs and indels were typed to perform GWAS on this population’s severity (lesion diameter) and tolerance grade. We used a compressed mixed linear model of TASSEL 5.0 software (Bradbury et al., 2003) for correlation analysis.

The threshold for the significance of tolerance grade was set at 0.05. Inputting the physical location of the SNP in the potato genome and its *P*-value, the qqman package of R software was used to draw the Manhattan plot and QQ plot. SAMtools was used to manually verify regions significantly correlated with the reordered read results of the potato reference genome PGSC_DM_v4.03.^7^

### Candidate Gene Screening

Based on potato SNP annotation and LD decay and according to the functional annotation of the loci, the genes on which the loci were located were used as candidate genes. If a locus was located upstream and downstream of other genes simultaneously, the upstream and downstream genes were also used as candidate genes. If a locus was located in the intergenic region, the upstream and downstream genes closest to the locus were used as candidate genes. A mixed linear model was used to perform association analysis on the traits of late blight.

### Real-Time Fluorescence Quantitative PCR Verification

Primers were designed based on the coding region sequence (CDS) of the candidate genes, and the actin gene was the internal reference (Table 2). A fluorescence reverse transcription kit (TaKaRa, Beijing) was used to generate cDNA using 500-ng RNA as the template. A fluorescence quantitative RT-PCR kit (TaKaRa, Beijing) was used to perform quantitative real-time polymerase chain reaction (qRT-PCR) with the thermal cycling program of 95°C for 30 s followed by 40 cycles of 95°C for 5 s, and 60°C for 30 s. Excel 2016 was used to sort and analyze the gene expression fluorescence qRT-PCR (quantitative real-time polymerase chain reaction) data. The 2^–ΔΔCt^ method was used to calculate the relative expression levels.

**TABLE 2:** qRT-PCR primers for the selected genes.

Gene ID	Forward primer (5′–3′)	Reverse primer (5′–3′)
Action	AGATGCTTACGCTGGATGGAATGC	TTCCGGTGTGGTTGGATTCTGTTC
PGSC0003DMG400000043	AGCAGCTCAAGCACAGAACTCTTC	CACGCTCCACCTCCAATTCATCTC
PGSC0003DMG400028682	TGGGCATCAACATTAACCCCAAA	CCAACCAGACAGGCTAGCCA

## Results

### Genotype Analysis of Potato Population

We obtained a total of 4,786,675 SNPs and indels in this study. All SNPs and indels were filtered for minor allele frequency (MAF) > 0.05 and Hardy–Weinberg equilibrium *P*-value > 0.001, and 20,382 high-quality SNPs and 2,107 indels were obtained. Annotation of the high-quality SNPs and indels showed that 18,683 (83.08%) were in intergenic regions; 3,806 (16.92%) were in the gene regions of the genome of which 951 were in the untranscribed areas, 2,796 were in introns, and only 1,682 SNPs were in the coding areas. In the coding regions, 771 SNPs produced silent mutations, and 911 SNPs produced missense mutations at a ratio of 1.18 (Table 3).

**TABLE 3:** Summary of single-nucleotide polymorphisms and indels.

Totals SNPs and indels	Intergenic	Untranslated region		Intron	Coding sequence			Non-syn/synratio
		3′ UTR	5′ UTR		Total	Missense	Synonymous	
22,489	18,683	538	413	2,796	1,682	771	911	1.18

### Identification and Analysis of the Late Blight Tolerance of Potato Leaves *in vitro*

Among the 284 materials tested, 37 resources with disease area less than 3% or asymptomatic and disease grade 1 were selected. The area of the disease spot was 3–10%, forming an anaphylactic dead spot; there was no chlorotic halo around it; and there were 15 resources with disease grade 2. There were 30 resources with disease grade 3 with white mycelium visible on the disease spot surface, and the area of the disease spot was 10–30%. The disease spot was water-soaked and surrounded by a chlorotic halo. There were 107 resources with disease grade 4, and the area of the disease spot was 30–60%. There were obvious chlorotic circles and white mold layers around the disease spot. There were 95 resources with disease grade 5; the lesion area was more than 60%, and a large amount of white mildew appeared on the lesion’s surface (Supplementary Table 2).

### Analysis of Population Structure

**FIGURE 1:**
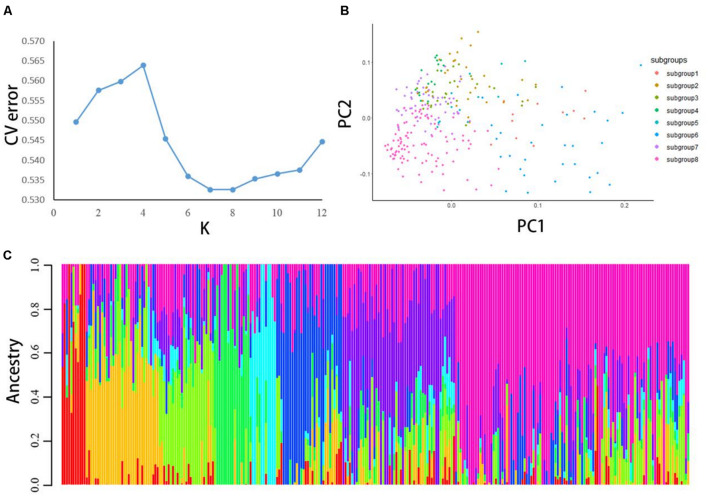
**(A)** The population structure of 284 potato materials was analyzed by using ADMIXTURE software. CV error was calculated when *K* = 1–12. **(B)** PCA was performed on all 284 potato samples with high-quality polymorphic loci. Each dot represents a sample. **(C)** When *K* = 8. In this population structure, each individual is represented by a line with eight different colors. According to the proportion of colors, which subgroup the variety belongs to can be inferred.

The ADMIXTURE software was used to analyze 22,489 high-quality SNPs and indels; the largest cluster subgroup value (K) was assumed to be each integer from 1 to 12, and the cross-validation (CV) error of each *K*-value was calculated (Figure 1A). When K was 1–4, the CV error gradually increased. When K was greater than 4, the CV error dropped rapidly to a nadir at *K* = 8, and for K > 8, it gradually increased. Therefore, *K* = 8 was optimal; that is, the entire potato population was divided into 8 subgroups.

PCA was conducted using all high-quality SNPs and indels. The calculation and analysis process was conducted by R software (Figure 1B). After the analysis was completed, plots were generated by R. For plotting, the eight subgroups inferred by the ADMIXTURE software were used for grouping. The results showed that the eight subgroups could be distinguished on the PC1 axis, and the clustering results were consistent with the population structure division.

According to the *Q*-value of each material in these eight subgroups, each material was classified into the subgroup with the largest *Q*-value (Figure 1C). Subgroups 1–8 had 11, 33, 25, 16, 12, 30, 51, and 86 germplasm resources, respectively. The distribution of the eight subgroups showed differences on the PC1 axis, and the clustering results were consistent with the population structure division. The eight subgroups of potatoes could not all be clustered together on the phylogenetic tree.

**FIGURE 2:**
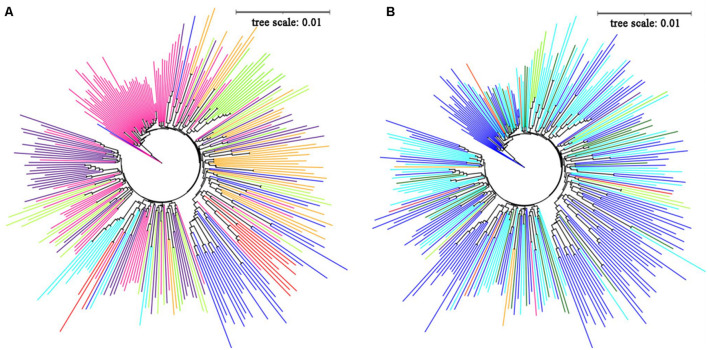
**(A)** The adjacent junction trees constructed by 284 potato materials, red, orange, green yellow, lawngreen, cyan, blue, indigo and deeppink represent subgroup 1-subgroup 8, respectively. **(B)** The source distribution of 284 potato materials in the evolutionary tree, red, orange, green yellow, lawngreen, cyan, blue, indigo, deeppink, orange red, violet represent Australia, Belarus, Britain, Canada, China, CIP, Israel, Netherlands, Russia, United States, darkgreen represent unknown sources.

The neighbor-joining method was used to construct a phylogenetic tree, and the tree diagram was drawn with iTOL software to explore the genetic relationships between the 284 potato germplasms. Overall, the clustering results were consistent with the division of the population structure: subgroups 1, 2, and 6 clustered together well, and samples of other subgroups could be clustered together, and there was a certain crossover between samples (Figure 2A). The results showed no significant relationship between the genetic relationship of potato germplasm and geographical origin (Figure 2B). Potatoes are native to the Andes of South America, and the history of artificial cultivation can be traced back to southern Peru from 8,000 to 5,000 BC.

### Genetic Diversity Revealed by Single-Nucleotide Polymorphism Markers

According to the 22,489 high-quality SNPs and indel data, the genetic diversity (π) of all 284 potato germplasm resources was 0.216, and the genetic diversity index of the eight subgroups was between 0.164 and 0.250. Among them, subgroup 8 had the lowest genetic diversity index (0.164), and 6 had the highest (0.250) (Table 4). These data show that there is rich genetic diversity in the 284 potato germplasm resources.

**TABLE 4:** Summary of genetic diversity (π).

Subgroup	1	2	3	4	5	6	7	8	Total
π	0.238	0.239	0.235	0.201	0.208	0.250	0.213	0.164	0.216

The pairwise population *F*-statistics (*F*_*ST*_), a measure of population differentiation, was used to evaluate the degree of difference between subgroups of the 284 potato germplasm resources (Table 5). It was found that the *F*_*ST*_ among the subgroups was between 0.025 and 0.149, and subgroups 1 and 8 had the highest *F*_*ST*_ (0.149) and subgroups 3 and 7 had the lowest *F*_*ST*_ (0.025). Subgroups 2 and 3, subgroups 2 and 7, subgroups 3 and 7, subgroups 3 and 8, and subgroups 7 and 8 were relatively weakly differentiated, and their genetic relationships were relatively close. In contrast, there was a moderate degree of differentiation between other subgroups.

**TABLE 5:** Summary of population pairwise *F*-statistics (*F*st).

Subgroup	1	2	3	4	5	6	7	8
1								
2	0.092							
3	0.088	0.029						
4	0.144	0.060	0.071					
5	0.131	0.080	0.072	0.123				
6	0.074	0.064	0.066	0.117	0.094			
7	0.108	0.033	0.025	0.061	0.073	0.087		
8	0.149	0.060	0.048	0.097	0.110	0.105	0.030	

### Genome-Wide Association Analysis of Late Blight Tolerance of Potato Germplasms

**FIGURE 3:**
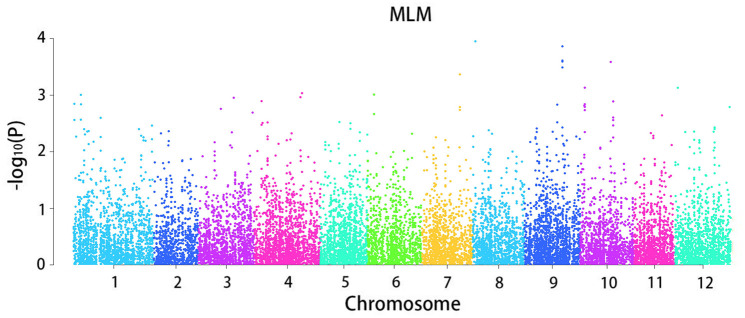
Manhattan plot of the whole-genome association analysis of disease grade.

**FIGURE 4:**
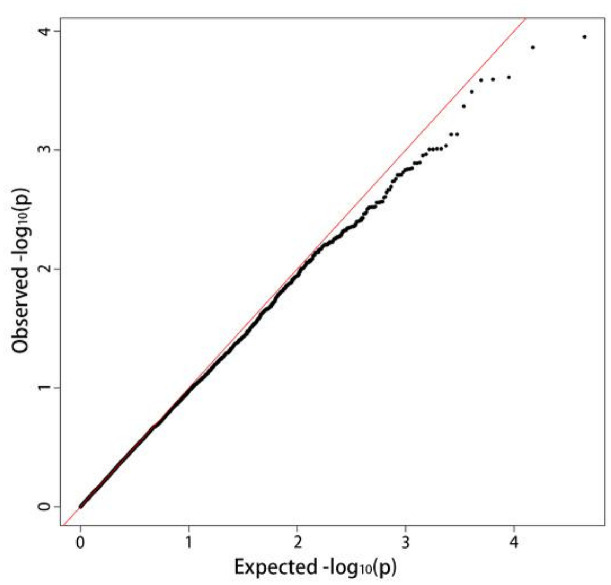
QQ plot of the whole-genome association analysis of disease grade.

The mixed linear model analysis performed correlation analysis on the disease tolerance grade (Figure 3). A total of 964 loci associated with late blight tolerance traits were identified (Supplementary Table 3). The results of the QQ map showed that the SNP sites associated with significant correlation were reliable (Figure 4). *P* < 0.05 was set as the threshold to determine the significant loci for the disease tolerance grade, and 14 candidate genes were obtained after annotating genes located at or near the significant loci.

### Potato Genome-Wide Linkage Disequilibrium

**FIGURE 5:**
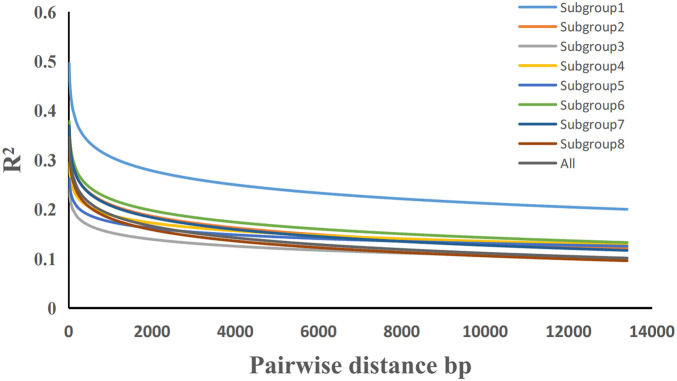
Genome-wide average linkage disequilibrium decay estimated from all potato samples.

The LD-decay curve was obtained by analyzing all 284 potato germplasm resources using the 22,489 SNP markers obtained from the whole genome (Figure 5). The results show that LD decreased as the physical distance between SNPs increased. When taking the coefficient of determination *R*^2^ = 0.2 as the decay threshold, the results show that LD decreases with increased physical distance between SNPs. When the coefficient of determination *R*^2^ = 0.2 is taken as the attenuation threshold. The attenuation distance from subgroups 1–8 was about 13.4, 1.3, 0.1, 0.7, 0.3, 1.9, 1.3, and 0.6 kb, respectively, and the attenuation distance of the whole population was about 0.9 kb. It was much lower than that of cultivated rice (123 kb), cultivated soybean (133 kb), and cultivated maize (30 kb), and slightly lower than that of cultivated cassava (8 kb) and a maize-inbred line population (1.5 kb) (Traut, 1994).

### Candidate Genes

According to the LD, we found 14 candidate genes. Most of the candidate genes were within 10 kb of the mutation site, and the largest was no more than 20 kb. Table 6 shows the detailed information. Among these genes, a gene encoding chitinase 1 was on chromosome 11 related to the immune response; six protein kinases, serine-threonine protein phosphatase, and mitogen-activated protein kinase genes were found around the significant sites on chromosomes 1, 4, 5, and 6. These protein kinases can catalyze protein phosphorylation and mediate signal transduction to external stimuli; on chromosomes 3 and 7, genes encoding ERF transcription factor, ethylene-responsive transcription factor 4, and ethylene receptor 2 were associated with ethylene response. Ethylene can coregulate with jasmonic acid pathogen invasions; on chromosomes 5, 6, and 8, the plant resistance protein, NBS-LRR tolerance protein, and late blight tolerance protein may be related to the immune response of late blight and the specific identification of pathogen effectors. NBS-LRR protein is the most important tolerance protein of late blight, which is directly related to the tolerance of potatoes to late blight. Additionally, one gene with unknown functions was on chromosome 6, requiring further verification.

**TABLE 6:** Candidate genes of significant association markers.

CHR	Position	Candidate gene	Start	End	Description
1	72,247,021	PGSC0003DMG400000043	72,233,966	72,243,677	Protein kinase
3	44,871,940	PGSC0003DMG400036493	44,865,282	44,866,451	ERF transcription factor
4	8,095,786	PGSC0003DMG400023584	8,093,725	8,096,358	Serine-threonine protein kinase
4	9,276,044	PGSC0003DMG400019737	9,274,796	9,280,465	Serine-threonine protein kinase
5	31,701,650	PGSC0003DMG400033661	31,687,765	31,689,237	Mitogen-activated protein kinase
5	3,253,2025	PGSC0003DMG400019926	32,513,930	32,514,640	Plant resistance protein
5	51,527,765	PGSC0003DMG400023346	51,518,931	51,524,033	Serine-threonine protein phosphatase
6	7,094,249	PGSC0003DMG400033671	7,082,039	7,082,791	Gene of unknown function
6	935787	PGSC0003DMG400031878	920,425	927,075	NBS-LRR resistance protein
6	39,855,777	PGSC0003DMG400016323	39,852,449	39854311	Serine-threonine protein kinase
7	49532127	PGSC0003DMG400026821	49,531,657	49,532,565	Ethylene-responsive transcription factor 4
7	51,816,228	PGSC0003DMG400027651	51,816,086	51,822,785	Ethylene receptor 2
8	11,363,730	PGSC0003DMG400047159	11,343,551	11,343,883	Late blight resistance protein
11	6,786,565	PGSC0003DMG400028682	6,788,959	6,789,991	Chitinase 1

### Expression Patterns of Candidate Genes

**FIGURE 6:**
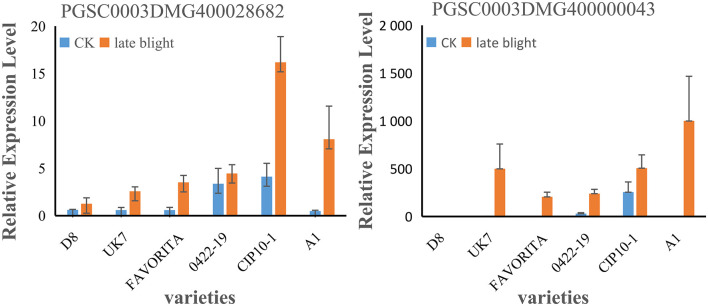
Experimental verification of gene expression levels by qRT-PCR.

Two candidate genes were randomly selected to verify their gene expression patterns in late blight tolerant varieties A1, CIP10-1, and 0422-19 and susceptible varieties D8, UK7, and FAVORITA by qRT-PCR. After inoculation with *Phytophthora infestans in vitro* for 5 days, the green leaves around the plaque and leaves without inoculation were used. Then, their relative expression levels were calculated (Figure 6). The results showed that most of the candidate genes were upregulated after inoculation. The expression levels of PGSC0003DMG400028682 in varieties that were tolerant were higher than those in susceptible varieties, and the expression levels of PGSC0003DMG400000043 in susceptible variety UK7 were higher than those in the tolerant variety 0422-19. The results showed that the tolerance to late blight was a quantitative trait controlled by multiple genes.

## Discussion

In our study, we evaluated the tolerance to *Phytophthora infestans* in 284 potato germplasms. Of these, 37 potato germplasms showed high tolerance. Population structure analysis assigned 284 potato germplasms into eight subpopulations. The high-quality SNP markers revealed a moderate level in the main differentiation among the eight subpopulations. Simultaneously, GWAS was conducted to identify the significant loci for late blight tolerance traits in germplasm resources and screen candidate genes.

### Identification and Analysis of Late Blight Tolerance of Germplasm Resources

In recent years, many reports present the identification of potato tolerance to late blight; however, there are some differences in the identification results. The mixed strains selected in this study have yet to be determined at the physiologic race level. With our studies for many years in the laboratory, we discovered that the tolerance of potato leaves to *Phytophthora infestans in vitro* was consistent with that at the adult stage. Therefore, the identification results of detached leaves could represent the tolerance at the adult stage. Favorita is a highly susceptible late blight variety, which is again consistent with our results. Identifying late blight-tolerant germplasm resources and discovering durable tolerant materials could be an important issue in breeding high-quality, late blight–tolerant potato varieties in the future. In this study, 37 materials showed immunity, and 15 germplasms conferred high tolerance to late blight disease, providing valuable materials for late blight-tolerant potato breeding.

Compared with identifying natural diseases in the field, *in vitro* leaf inoculation identification has certain advantages and accuracy. First, the identification of *in vitro* leaf inoculation is indoor; the temperature and humidity conditions are controllable, which can create the most suitable conditions for the occurrence of late blight. The identification results are more accurate, avoiding the error caused by human factors in identifying natural diseases in the field. Additionally, the physiological races in the Qinghai Province of Northwest China were used to identify the tolerance to late blight for 2 years. Breeding tolerant varieties is the most economical and effective means to prevent and control late blight. The screening and utilization of tolerant resources is the basis and core of disease-tolerance breeding. In this study, *in vitro* leaf inoculation identification can identify its tolerance to late blight earlier than potato tuber inoculation identification, and the incidence can be clearly seen. Additionally, *in vitro* leaf inoculation identification can avoid releasing the late blight pathogen into the environment, causing a large area of late blight or becoming a potential risk.

### Analysis of Potato Population Structure and Genetic Diversity

In this study, 284 potato germplasm resources were used for the association analysis of the tolerance to late blight, 138 of which were from the CIP. By testing genetic markers for Hardy–Weinberg equilibrium and suballelic frequency filtering, 22,489 high-quality polymorphic loci were obtained. The stratification of population structure and the uneven distribution of alleles are important reasons for false associations between genotypes and traits (Flint-Garcia et al., 2003). The population structure analysis of 284 potato germplasm resources in this study found that, when *K* = 8, the CV error value was the smallest. Therefore, 284 potato germplasm resources were divided into eight subgroups. The genetic diversity index of the potato population was 0.216, indicating that there was abundant genetic diversity. The *F*_*ST*_ among subgroups was mostly between 0.05 and 0.15, and the differentiation between subgroups was mostly moderate, indicating a certain degree of differentiation in germplasm resources. Still, the degree of differentiation was not high.

### Analysis of Candidate Genes

The reference genome is a diploid potato reference genome in this study, and no tetraploid genome existed. Through GWAS, significant variation sites are mined to reasonably analyze and mine candidate genes. The SNPs in annotated loci were obtained by combining the SNP genomic positions (Maria et al., 2017). In the future, if there is a better four ontology reference genome, we will fully consider LD, and then more detailed and accurate mining of candidate genes.

Late blight is a serious disease worldwide, threatening the potato industry and food security. Late blight-tolerance potato breeding is very important in China and abroad, and breeding to have multiple disease-tolerance genes in the same variety is important for preventing late blight. So far, 11 broad-spectrum tolerance genes (R1–R11) have been discovered, and these 11 major R genes have been successfully located on the potato genetic map (Li et al., 1998; Ballvora et al., 2002). Combinations of different disease-tolerance genes can provide ideal late blight tolerance (Rietman et al., 2012). Therefore, optimizing the known disease-tolerance gene combinations, making full use of R disease-tolerance genes with broad-spectrum tolerance characteristics, and discovering new broad-spectrum and longer-lasting disease-tolerance genes from the abundant potato resources are effective means for cultivating tolerant potato varieties in the future (Haverkort and Hillier, 2011). The abundant wild resources of potatoes are the source of R genes. Potato contains many genes encoding cytosolic NBS-LRR tolerance proteins (Jupe et al., 2012). The R gene can be introduced into cultivated varieties by crossing conventional varieties with wild species containing this gene, thus helping cultivate varieties to achieve durable tolerance to late blight (Halterman et al., 2008). In the abundant potato resources, many unknown late blight-tolerance genes are waiting to be discovered.

In this study, 19 candidate genes, respectively, were found by the GWAS of the disease severity grade identified from isolated potato leaves affected by late blight *in vitro*. Among them, the candidate gene encoding the chitinase 1 gene may be involved in the immune response, and chitinase can improve the tolerance of plants to fungi (Hiroshi et al., 1997; Tabei et al., 1998). The GWAS of disease tolerance grade found that three candidate genes were associated with ethylene response. Ethylene could coordinate with jasmonic acid to regulate plant immunity during pathogen invasion (Lieselotte et al., 2014). Two resistance proteins and one NBS-LRR tolerance protein may be directly related to potato late blight tolerance by the correlation analysis of the disease-tolerance grade. NBS-LRR proteins are the most critical late blight tolerance proteins, characterized by the same conserved structure containing an N-terminal leucine zipper or coiled-coil, a nucleotide-binding site, and leucine-rich repeats. The cloned anti–late blight genes have several highly conserved structures, such as a phosphate-binding domain (Ploop), a kinase-2 group, or a GLPL group in the NBS region (Traut, 1994; Meyers et al., 1999). This study provides genetic resources for follow-up research and lays the foundation for genetic improvement of potato tolerance to late blight.

## Data Availability Statement

The original contributions presented in the study are publicly available. This data can be found here: https://ngdc.cncb.ac.cn/search/?dbId=bioproject&q=PRJCA005945&page=1, PRJCA005945.

## Author Contributions

FW, ZX, and JW conceived and designed the experiments. FW and LZ conducted the experiments work. ZX, MZ, and LZ analyzed the experimental results. FW and MZ wrote the manuscript. ZX and JW reviewed and contributed to improve it and revised the last version of the manuscript. All authors read and approved the final manuscript.

## Conflict of Interest

The authors declare that the research was conducted in the absence of any commercial or financial relationships that could be construed as a potential conflict of interest.

## Publisher’s Note

All claims expressed in this article are solely those of the authors and do not necessarily represent those of their affiliated organizations, or those of the publisher, the editors and the reviewers. Any product that may be evaluated in this article, or claim that may be made by its manufacturer, is not guaranteed or endorsed by the publisher.

## Footnotes

1
http://afsmseq.sourceforge.net/

2
http://solanaceae.plantbiology.msu.edu/

3
http://vcftools.sourceforge.net/

4
http://evolution.genetics.washington.edu/phylip.html

5
https://itol.embl.de/

6
https://vcftools.github.io/index.html

7
http://solanaceae.plantbiology.msu.edu/pgsc_download.shtml/

## Supplementary Material

Supplementary Table 1 The 284 potato germplasm resources used for GWAS.

Supplementary Table 2 Identification and analysis of the late blight tolerance of potato leaves *in vitro.*

Supplementary Table 3 Significant variation sites.
